# How to resume safe access to a medical simulation center at the time of COVID-19 pandemic: The proposal of a protocol from a university institution in North-Eastern Italy

**DOI:** 10.30476/JAMP.2021.90616.1418

**Published:** 2022-01

**Authors:** Carlo Biddau, Alessandro Tel, Pier Paolo Brollo, Massimo Robiony, Vittorio Bresadola

**Affiliations:** 1 General Surgery Department and Simulation Center, Academic Hospital of Udine, Department of Medicine, University of Udine, Italy; 2 Maxillofacial Surgery Department, Academic Hospital of Udine, Department of Medicine, University of Udine, Italy

**Keywords:** Continuing education, Teaching, SARS-CoV-2, Academic Medical Centers

## Abstract

**Introduction:**

The Covid-19 global pandemic has suspended thousands of clinical education programs around the world. Also in Italy, as in the rest of the world, frontal teaching activities and internships in the medical field have been suspended. At the university hospital of Udine (North-Eastern Italy) it was decided to strengthen the use of simulation in all training stages to get over the block of training activities.

**Methods:**

A protocol has been drawn up with the aim of providing training in safety for every student of the degree courses in medicine and health area and for doctors in residency training. In this way it was possible to carry out training sessions with a maximum of 6 students engaged in the simulation activities offered by the Center (3D) virtual cadaver, laparoscopic pelvic trainer stations, ultrasound laboratory, microsurgery, etc.). The key points of the protocol were represented by i) internet booking of the training activity; ii) respect of safety measures (hand hygiene, safe distance, restricted total number of presences, constant use of the surgical mask) and iii) reorganization of the material and cleaning of the rooms.

**Results:**

Our educational strategy allowed to resume training activity maintaining adequate levels of safety for students and teachers. Applying our protocol, it was possible to guarantee safe access to our Medical Simulation Center (MSC) to a total of about 1400 students from different course of study during the period between June 2020 and February 2021.

**Conclusions:**

Our protocol could represent a practical tool in the management of resuming the activity at a MSC.

## Introduction

In Italy since the beginning of March 2020, with the rapid spread of the Covid-19 pandemic, all university frontal training activities in the medical and health field have been blocked, with a concurrent (sometimes complete) restriction on the attendance of teaching hospitals. Only postgraduates have continued to attend their clinical training programs. 

In a short time (weeks), to overcome this, new training strategies have been activated such as the use of multimedia platforms, smart learning or tele-mentoring. To date, it is unclear when it will be possible to resume traditional training activities such as classroom lessons or group internships in the hospital. Currently, in Italy it is believed that the complete resumption of frontal teaching will take place with the spread of the vaccine phase ( 1 ). In recent months, this educational problem has appeared in all the countries where the pandemic has spread, leading to the suspension of thousands of clinical education programs around the world ( 2 ). 

At the university hospital of Udine, in addition to the strategies mentioned above, it was decided to strengthen the use of simulation in all training stages (pre- post graduate education, continuous training). In support of this, in Italy, the Minister of University and Research, with the Ministerial decree (MD) n. 58 of 29 April 2020, indicates in the simulation a method for the laboratory activities in order to obtain the academic qualification, maintaining adequate training standards without exposing students to the risk of virus transmission ( 3 ). 

As we know, simulation is an educational technique that replaces or amplifies real experiences with guided experiences that evoke or replicate the substantial aspects of the real world in a fully interactive way ( 4 ). In the medical-health field, this technique does not replace clinical training “at the patient’s bed” but, by integrating with the latter, it allows a faster acquisition of technical and non-technical skills, in complete safety. In the pandemic era, the simulation technique becomes a further safe training tool, reducing the risk of spreading Covid-19 infection in the academic community and clinical traineeship environments ( 5 ). In fact, simulation can be done alone (trainer for sutures, for example) or in limited groups such as in high fidelity simulation, avoiding contact with patients and assemblages. In this critical phase related to the Covid-19 pandemic, we are faced with practical and logistical challenges and concerns for patient safety, recognizing that students may potentially spread the virus when asymptomatic and may acquire the virus in the course of training ( 6 ).

On the other hand, the reopening of MSC, closed during the lockdown of the pandemic, can be complex in the absence, in literature, of national and/or international guidelines. 

At the University of Udine there is a simulation and advanced training center (CSAF) ( 7 ) situated in the teaching hospital used for pre- and postgraduate and continuing education in the medical and health field. The center is partly organized as a virtual hospital (Operating theatre, Intensive care unit, Hospitalization, Radiology room) to which spaces are associated for surgical training and self-managed training. The total space is about 800 m ( 2 ). In the absence of institutional guidelines or scientific societies, as was the case in other areas of medical activity, such as for example the resumption of surgical activity ( 8 ), a protocol has been drawn up with the aim of providing training in safety and respecting ministerial indications provided by the Italian Government, the reports of the Istituto Superiore di Sanità ( 9 ), the main technical-scientific entity of the Italian Health Service, the ordinances of the FVG region and the documentation provided by the clinical risk service of the University Hospital of Udine in the months of April-May 2020 ( 9 , 10 ). Our committee worked with experts from other specialties (infectious disease specialist, Hygienist and Public Health Specialist) to develop this guidance in accordance with World Health Organization’s (WHO) instructions ( 11 ). The hospital management endorsed the protocol before it was used since 10 June 2020.

**Figure 1:**
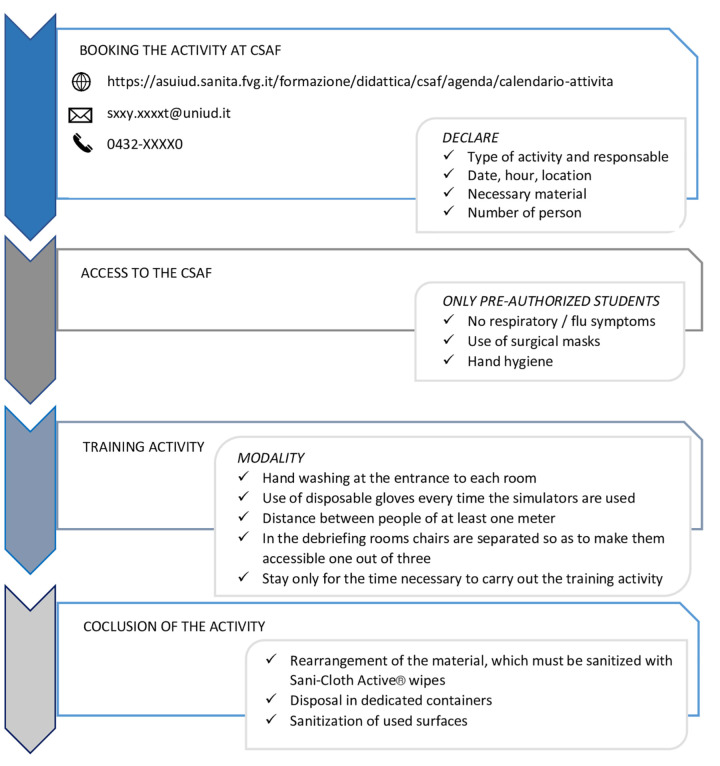
The essential points of the protocol

The aim of this report is to share the fundamental points of the protocol believing that it could be, in the lack of specific guidelines, a practical guide for other MSC in the management of resuming their activity and in the knowledge of the incoming related problems, after the lockdown period. The essential points are summarized in Figure 1.

## The protocol

### Who can enter the simulation center?

All students of the degree courses in medicine and health area, doctors in residency training and their tutors can have access to the center, but patients (standardized patients also) and external visitors do not. 

*Goal: to allow the access only to those who have a training need for curricular progression.*

### Booking the activity

Access to the MSC must be preceded by a booking and subsequent confirmation by the manager of the training activity. The booking must be made using the annual planning on the Center's website or by telephone directly contacting the secretary of the Center. For the booking it is necessary to indicate the number of people who will be present, the name, birth’s data and telephone number of the contact person, the type of activity (low or high-fidelity simulation, etc.) and possibly the material to be prepared in collaboration with the technical-administrative staff of the MSC.

*Goal: to be able to track persons entering the center. To minimize their stay during the simulation session.*

### Access

It is possible to access the MSC only for all persons previously authorized. People with symptoms (especially fever, cough, dyspnea, diarrhea, anosmia, dysgeusia) are not allowed to access the center. At the entrance of the MSC, it is necessary to perform hand hygiene using the hydroalcoholic solution dispenser as recommended by the WHO ( 12 ). All visitors must use the surgical mask. In order not to create gathering situations, the number of participants for a single training session must be a maximum of 6 people, excluding the support staff allocated in dedicated spaces (secretary, direction). The total number of presences can be a maximum of 10 if the activity is organized individually with a trainer per learner and in different rooms. Every room can host a maximum of three trainers per session.

Access to the toilets and changing rooms is organized individually, the introduction of personal effects (bags, backpacks, umbrellas, etc.) must be kept to a minimum. Company uniforms are available to be used only during the stay at the Center. 

*Goal: Avoid access of Covid-19 positive suspects and gatherings during training activities. Respect of hygiene regulations.*

### Carrying out the training activity and suggestions

The independent attendance (single person) of the MSC (as in the case of simulation in ophthalmic surgery, microsurgery, laparoscopy and surgical planning) always requires compliance with the prevention and control measures of the infection, performing hand hygiene at the entrance and at the exit of the room together with the constant use of the surgical mask. 

The MSC is organized into four main areas: an Operating theatre, an Intensive care unit, an Inpatient ward and a Radiology room. In each area there are specific trainers to conduct different simulation workshops. Students engaged in operating theatre will train with laparoscopic, ophthalmic and suturing trainers. Intensive care unit laboratory is equipped with different arterial and venous access simulators and pediatric and adult airway management training manikins. In the Inpatient unit there are different trainers such as ATLS (Advanced Life Trauma Support) thorax drainage kit, cardiac auscultation simulator, manikins for nasogastric tube positioning and intramuscular and intraosseous injection simulators. Finally, the radiology area is furnished with a three-dimensional anatomy platform, a simulator of radiological errors, and a three-dimensional printer ( 13 ).

During team activities it is necessary to comply with the rule of distancing between people of at least one meter. Hand hygiene must be performed every time the environment is changed within the center. As for the use of the debriefing rooms, it is necessary to space the seating as follows: if there are several rows of chairs, it is necessary to guarantee the safety distance, by leaving two empty chairs in between occupied seats and leaving an empty seat at the front and the rear of the occupied chair. It is mandatory to stay at the MSC only for the strictly necessary time periods. Other related training phases can be requested or anticipated for example using remote platforms. The training session at the MSC can be amplified in terms of use by using teleconferencing platforms. In this case, those present must complete the appropriate consent form. The teachers use the spaces and equipment of the Center to produce videos useful for strengthening the teaching activity remotely.

*Goal: stress behavioral rules such as distancing and reduce the time spent in the center by encouraging the complementary use of remote platforms.*

### Reorganization of the material and cleaning of the rooms

At the end of each session, the training manager must reorder the material, which must be sanitized with Sani-Cloth Active^®^ wipes, and the disposal of everything that is not reusable in specific services. At the end of the activity, users of the Center must sanitize their personal surfaces (desk, chair, keyboard, PC, mouse, telephones, handles, pens, etc.) using the wipes indicated above. The cleaning and sanitization of the premises is weekly. In exceptional cases it is possible to request a supplementary intervention.

*Goal: share with those who use the center its security in terms of cleanliness and disinfection.*

## Results

Starting from the month of May 2020, parallel to the reduction of restrictive rules correlated to the national lockdown that had led to the suspension of simulation activities in March and April, the application of our protocol has allowed the reopening of the simulation center.

Between May 2020 and January 2021, it was possible to guarantee safe access to our MSC to a total of about 1400 people between residents and students from different courses of studies. More than 210 groups and 140 single access sessions were carried on. Despite a continuous reduction in the number of activities compared to the pre-pandemic year 2019 (5772 accesses), the resumption of the simulation’s activity has allowed, in the degree courses of medicine, nursing and obstetrics, the acquisition of clinical skills that were usually acquired in internships in hospital divisions. Frequency was almost completely suppressed in 2020 due to the pandemic. 

Regarding postgraduate training, these months have seen an increase in the presence of doctors training at the center, especially for surgical disciplines. In fact, even though residents were able to attend the hospital’s departments, there has been a significant reduction in their clinical and surgical activities. At the center, young surgeons have been able to acquire and maintain skills using the laparoscopic platform, the augmented reality simulator for ophthalmic surgery, the virtual anatomical table and the software for preoperative surgical planning ( 14 ). 

The impossibility to organize events in presence has facilitated the introduction of teleconference meetings. The staff of pediatrics of our hospital has organized, with regular cadences, simulations in remote, connecting through the web with other university centers in Europe and North America. These modalities will also be maintained in the post pandemic, for their potentiality and formative efficacy.

## Conclusion

The pandemic has disrupted traditional training paradigms. In Italy, after the declaration of the lockdown, there has been a rapid and efficient transformation of the educational system (at all levels) with a massive use of distance learning. In the medical and health care fields this is a limiting factor because it does not allow the acquisition of technical abilities or skills related to decision making or team working. Simulation is a technique that allows to do all this and, in this period of restrictions for pandemics, it should be used more intensively and frequently. From this point of view, the opening of simulation centers is a fundamental step to ensure pre- and postgraduate curricular progression.

Our proposed strategy is designed to help MSCs to guarantee the medical education in a well-balanced, practical and safe manner during the COVID-19 outbreak. This strategy may be subject to future adjustments and adaptation corresponding to new developments in the COVID-19 outbreak.

Finally, we are aware that this protocol is a set of behavioral indications that will have to be adapted to each individual situation according to the specific rules already existing in the individual countries and the characteristics of the individual simulation center.

## Conflict of Interest:

None Declared. 
